# Supplementary material for: FishResp: R package and GUI application for analysis of aquatic respirometry data
Source: Conserv Physiol. 2019 Feb 6;7(1):coz003. doi: 10.1093/conphys/coz003 (PMC6364290; doi:10.1093/conphys/coz003)
Supplement: Supplementary Data [file coz003_supplementary_revised.docx]

**Supplementary files (R scripts)**

**File S1.** R script for running the stickleback example. Comments and information about the scripts (not run) are preceded by a hash symbol (#) and shaded in grey. Our thanks to an anonymous reviewer of a previous draft who provided insightful and helpful commentary of the code which we have also incorporated here.

# # # # # # # # # # # # # # # # # # # # # # # # # # # # # # # # # # # # #

# Species: Gasterosteus aculeatus (three-spined stickleback) #

# Chamber info: AutoResp, n=4, V=250ml #

# Trait.1: Standard metabolic rate (SMR) #

# Trait.2: Active metabolic rate (AMR) #

# Background respiration: pre and post #

# # # # # # # # # # # # # # # # # # # # # # # # # # # # # # # # # # # # #

# Loading the package 'FishResp' into R environment

library(FishResp)

# Specifying paths to the files containing raw respirometry data for Case Study 1

pre.path = system.file("extdata/stickleback/pre_raw.txt.xz", package = "FishResp")

post.path = system.file("extdata/stickleback/post_raw.txt.xz", package = "FishResp")

SMR.path = system.file("extdata/stickleback/SMR_raw.txt.xz", package = "FishResp")

AMR.path = system.file("extdata/stickleback/AMR_raw.txt.xz", package = "FishResp")

# Filling the information about animals, respirometry chambers and DO units

info <- input.info(ID = c("Stickleback_1", "Stickleback_2",

"Stickleback_3", "Stickleback_4"),

Mass = c(1.86, 1.92, 2.23, 1.80),

Volume = c(250, 250, 250, 250),

DO.unit = "mg/L")

# Note, only three DO units are available in FishResp: "mg/L", "mmol/L", or "ml/L".

# Other DO units should be converted to FishResp compatible ones using the functions

# 'convert.respirometry' or 'convert.rMR' (see File S3 and the R package documentation)

# Importing background respiration tests (pre and post)

pre <- import.test(pre.path,

info.data = info,

logger = "AutoResp",

n.chamber = 4,

plot.temperature = TRUE,

plot.oxygen = TRUE)

post <- import.test(post.path,

info.data = info,

logger = "AutoResp",

n.chamber = 4,

plot.temperature = TRUE,

plot.oxygen = TRUE)

# Importing raw data for SMR and AMR measurements.

SMR.raw <- import.meas(SMR.path,

info.data = info,

logger = "AutoResp",

n.chamber = 4,

date.format = "DMY",

start.measure = "22:00:00",

stop.measure = "06:00:00",

plot.temperature = TRUE,

plot.oxygen = TRUE)

AMR.raw <- import.meas(AMR.path,

info.data = info,

logger = "AutoResp",

n.chamber = 4,

date.format = "DMY",

plot.temperature = TRUE,

plot.oxygen = TRUE)

# Correcting raw data for background respiration

SMR.clean <- correct.meas(info.data = info,

pre.data = pre,

meas.data = SMR.raw,

method = "pre.test")

AMR.clean <- correct.meas(info.data = info,

post.data = post,

meas.data = AMR.raw,

method = "post.test")

# QC graphical tests of raw data before and after correction

# Here, we recommend maximizing a plot window in R

QC.meas(SMR.clean, "Temperature")

QC.meas(SMR.clean, "Total.O2.phases")

QC.meas(SMR.clean, "Corrected.O2.phases")

QC.meas(SMR.clean, "Total.O2.chambers")

QC.meas(SMR.clean, "Corrected.O2.chambers")

QC.meas(AMR.clean, "Temperature")

QC.meas(AMR.clean, "Total.O2.phases")

QC.meas(AMR.clean, "Corrected.O2.phases")

QC.meas(AMR.clean, "Total.O2.chambers")

QC.meas(AMR.clean, "Corrected.O2.chambers")

# Activity of animals during SMR measurements

QC.activity(SMR.clean, compare = FALSE)

# Mass-specific metabolic rate before and after correction for background

# respiration (note, metabolic rate is calculated without any slope filtration)

QC.activity(SMR.clean, compare = TRUE)

# Extraction of target slopes for SMR and AMR

SMR.slope <- extract.slope(SMR.clean,

method = "min",

n.slope = 3,

r2 = 0.95,

length = 1200)

AMR.slope <- extract.slope(AMR.clean,

method = "all",

r2 = 0.95,

length = 600)

# Detailed graphical description of extracted slopes for both SMR and AMR

QC.slope(SMR.slope, SMR.clean, chamber = "CH1", current = 1200, alter = 600)

QC.slope(SMR.slope, SMR.clean, chamber = "CH2", current = 1200, alter = 600)

QC.slope(SMR.slope, SMR.clean, chamber = "CH3", current = 1200, alter = 600)

QC.slope(SMR.slope, SMR.clean, chamber = "CH4", current = 1200, alter = 600)

QC.slope(AMR.slope, AMR.clean, chamber = "CH1", current = 600, alter = 300)

QC.slope(AMR.slope, AMR.clean, chamber = "CH2", current = 600, alter = 300)

QC.slope(AMR.slope, AMR.clean, chamber = "CH3", current = 600, alter = 300)

QC.slope(AMR.slope, AMR.clean, chamber = "CH4", current = 600, alter = 300)

QC.slope(AMR.slope, AMR.clean, chamber = "CH1",

current = 600, alter = 300, residuals = TRUE)

QC.slope(AMR.slope, AMR.clean, chamber = "CH2",

current = 600, alter = 300, residuals = TRUE)

QC.slope(AMR.slope, AMR.clean, chamber = "CH3",

current = 600, alter = 300, residuals = TRUE)

QC.slope(AMR.slope, AMR.clean, chamber = "CH4",

current = 600, alter = 300, residuals = TRUE)

# Reducing the length of measurements for each AMR slope

AMR.slope <- extract.slope(AMR.clean,

method = "all",

r2 = 0.95,

length = 300)

# Calculation of background respiration rate, absolute and

# mass-specific metabolic rate for SMR and AMR

SMR <- calculate.MR(SMR.slope,

density = 1000,

plot.BR = TRUE,

plot.MR.abs = TRUE,

plot.MR.mass = TRUE)

AMR <- calculate.MR(AMR.slope,

density = 1000,

plot.BR = TRUE,

plot.MR.abs = TRUE,

plot.MR.mass = TRUE)

# Exporting the final dataset as a .txt file: SMR and AMR datasets

# are merged into one dataframe. In addition, absolute,

# mass-specific and factorial metabolic rate are calculated.

results <- export.MR(SMR, AMR,

file = "results.txt",

simplify = TRUE,

MS = TRUE,

plot.MS.abs = TRUE,

plot.MS.mass = TRUE,

plot.MS.fact = TRUE)

########### The end ###########

**File S2**. R script for running the guppy example (Case Study 2)

# # # # # # # # # # # # # # # # # # # # # # # # # # # # #

# Species: Poecilia reticulata (guppy) #

# Chamber info: AutoResp, n=8, V=21.8 ml #

# Trait: Standard metabolic rate (SMR) #

# Background respiration: pre and post #

# # # # # # # # # # # # # # # # # # # # # # # # # # # # #

# Loading the package 'FishResp' into R environment

library(FishResp)

# Specifying paths to the files containing raw respirometry data for Case Study 2

pre.path = system.file("extdata/guppy/pre_raw.txt.xz", package = "FishResp")

post.path = system.file("extdata/guppy/post_raw.txt.xz", package = "FishResp")

SMR.path = system.file("extdata/guppy/SMR_raw.txt.xz", package = "FishResp")

# Filling the information about animals, respirometry chambers and DO units

info <- input.info(ID = c("Guppy_M1", "Guppy_M2", "Guppy_M3", "Guppy_M4",

"Guppy_F1", "Guppy_F2", "Guppy_F3", "Guppy_F4"),

Mass = c(0.097, 0.086, 0.065, 0.090, 0.253, 0.428, 0.181, 0.222),

Volume = c(21.8, 21.8, 21.8, 21.8, 21.8, 21.8, 21.8, 21.8),

DO.unit = "mg/L")

# Importing background respiration tests (pre and post)

pre <- import.test(pre.path,

info.data = info,

logger = "AutoResp",

n.chamber = 8,

plot.oxygen = TRUE,

plot.temperature = TRUE)

post <- import.test(post.path,

info.data = info,

logger = "AutoResp",

n.chamber = 8,

plot.oxygen = TRUE,

plot.temperature = TRUE)

# Importing raw data for SMR measurements.

SMR.raw <- import.meas(SMR.path,

info.data = info,

logger = "AutoResp",

n.chamber = 8,

start.measure = "22:00:00",

stop.measure = "06:00:00",

plot.oxygen = TRUE,

plot.temperature = TRUE)

# Correcting raw data for background respiration

SMR.clean <- correct.meas(info.data = info,

pre.data = pre,

post.data = post,

meas.data = SMR.raw,

method = "exponential")

# QC graphical tests of raw data before and after correction

# Here, we recommend maximizing a plot window in R

QC.meas(SMR.clean, "Temperature")

QC.meas(SMR.clean, "Total.O2.phases")

QC.meas(SMR.clean, "Corrected.O2.phases")

QC.meas(SMR.clean, "Total.O2.chambers")

QC.meas(SMR.clean, "Corrected.O2.chambers")

# Activity of animals during SMR measurements

QC.activity(SMR.clean, compare = FALSE)

# Mass-specific metabolic rate before and after correction for background

# respiration (note, metabolic rate is calculated without any slope filtration)

QC.activity(SMR.clean, compare = TRUE)

# Extraction of target slopes for SMR

SMR.slope <- extract.slope(SMR.clean,

method = "calcSMR.quant",

p = 0.25, r2 = 0.9)

# Note, if one of the ‘calcSMR’ methods has been used, a detailed graphical

# description of extracted slopes will not be available. To be able to run graphical

# QC tests for corrected raw data with fitted linear models, all slopes should be

# extracted and plotted before applying the ‘calcSMR.quant’ method. This

# algorithm is demonstrated below (optional).

SMR.slope <- extract.slope(SMR.clean, method = "all", r2 = 0.9)

QC.slope(SMR.slope, SMR.clean, chamber = "CH1") # same for other chambers…

SMR.slope <- extract.slope(SMR.clean,

method = "calcSMR.quant",

p = 0.25, r2 = 0.9)

# Calculation of background respiration rate, absolute and

# mass-specific metabolic rate for SMR

SMR <- calculate.MR(SMR.slope,

density = 1000,

plot.BR = TRUE,

plot.MR.abs = TRUE,

plot.MR.mass = TRUE)

# Exporting the final dataset as a .csv file.

results <- export.MR(SMR, file = "results.csv", simplify = TRUE)

########### The end ###########

**File S3.** R script demonstrating application of functions listed in alphabetical order which have not been reviewed in the case studies.

# Example of converting oxygen units using the function 'convert.respirometry'

SMR.path = system.file("extdata/stickleback/SMR_raw.txt.xz", package = "FishResp")

convert.respirometry(import.file = SMR.path,

export.file = "converted_SMR_raw.txt",

n.chamber = 1, logger = "AutoResp",

from = "mg_per_l", to = "mmol_per_l",

sal = 0, atm_pres = 1013.25)

# Example of converting oxygen units using the function 'convert.rMR'

AMR.path = system.file("extdata/stickleback/AMR_raw.txt.xz", package = "FishResp")

convert.rMR(import.file = AMR.path,

export.file = "converted_AMR_raw.txt",

n.chamber = 2, logger = "AutoResp", salinity = 0,

DO.units.in = "mg/L", DO.units.out = "PP",

bar.press = 101.325, bar.units.in = "kpa")

# Example of importing raw data recorded by 'Q-box Aqua' using the function 'import.meas'

qbox.path = system.file("extdata/qboxaqua/qboxaqua.csv", package = "FishResp")

info <- input.info(ID = "Fish_1", Mass = 2.3, Volume = 170, DO.unit = "mg/L")

RMR.raw <- import.meas(file = qbox.path,

info.data = info,

logger = "QboxAqua",

n.chamber = 1,

date.format = "DMY",

start.measure = "23:30:00",

stop.measure = "01:00:00",

set.date.time = "23/02/2014/23:30:22",

meas.to.wait = 200,

plot.temperature = TRUE,

plot.oxygen = TRUE)

# Example of converting raw data from 'OxyView' (PreSens) and a summary file from

# 'AquaResp' (free software) to 'FishResp' format using the function 'presens.aquaresp'

presens.path.1 = system.file("extdata/presens/presens-ch1.txt", package = "FishResp")

presens.path.2 = system.file("extdata/presens/presens-ch2.txt", package = "FishResp")

presens.path.3 = system.file("extdata/presens/presens-ch3.txt", package = "FishResp")

presens.path.4 = system.file("extdata/presens/presens-ch4.txt", package = "FishResp")

aquaresp.path = system.file("extdata/presens/presens-aquaresp.txt", package = "FishResp")

presens.aquaresp(presens.file = c(presens.path.1, presens.path.2,

presens.path.3, presens.path.4),

aquaresp.file = aquaresp.path,

fishresp.file = "fishresp.txt",

date.format = "DMY",

n.chamber = 4,

wait.phase = 60,

measure.phase = 240)

# Example of converting raw data from 'Pyro Oxygen Logger' (PyroScience) and

# a summary file from 'AquaResp' (free software) to 'FishResp' format using

# the function 'pyroscience.aquaresp'

pyroscience.path = system.file("extdata/pyroscience/pyroscience.txt", package = "FishResp")

aquaresp.path = system.file("extdata/pyroscience/pyroscience-aquaresp.txt",

package = "FishResp")

pyroscience.aquaresp(pyroscience.file = pyroscience.path,

aquaresp.file = aquaresp.path,

fishresp.file = "fishresp.txt",

date.format = "MDY",

n.chamber = 1,

wait.phase = 120,

measure.phase = 600)

# Example of removing poor quality data using the function 'rm.data'

data(AMR.clean)

AMR.clean.modified <- rm.data(AMR.clean,

chamber = "CH3",

M.phase = c("M1","M2"))

###################### The end ###########################
